# Opium Smoking

**Published:** 1879-04

**Authors:** R. C. Hamill

**Affiliations:** 629 Wabash av.


### ARTICLE XIX.

### OPIUM SMOKING.

*Dear Sir:* I enclose you a brief article on opium smoking. It was published in the *Missionary Herald*, January, 1879, the organ of the American Board of Commissioners for Foreign Missions, and is altogether reliable.

R. C. HAMILL, 629 Wabash av.

February 10, 1879.

### OPIUM ASYLUM AT FOOCHOW.—TREATMENT.

Can the victim of opium smoking be rescued? It is commonly believed that, so terrible is his appetite, he is beyond help and hope. The asylum at Foochow has demonstrated the falsity of this assertion. Connected with the hospital of our mission, under charge of Dr. Osgood, there is a separate building for the treatment of opium smokers, where about sixty patients are received each month. In response to an inquiry as to the method of treatment, Dr. Osgood sends the following account of his practice for the last two years, during which time about 800 cases have been treated:

"1st. The total and absolute discontinuance of the use of opium from the beginning of treatment.

"2d. A trusty attendant to be with the patient, day and night, for the first three days.

"3d. Chloral hydrate for the first three nights, if required.

"4th. Good food, milk, raw eggs, brandy (in some cases), chicken broth. (The above to be taken in small quantities and frequently.

"5th. In diarrhœa, give two drachm doses of a mixture of equal parts of tincture catechu and tincture ginger.

"6th. Vomiting will frequently yield to bismuth in fifteen grain doses; and in some cases a single dose of calomel has acted like a charm. Ice would be of advantage in some cases.

"7th. Throughout the entire treatment it should be remembered that the patient is below par, and requires tonics. Quinine and tincture of iron have a prominent place in our list.

"8th. The patient should expect to suffer more or less for the first three days, and should make himself a prisoner for that time. By the fourth day there is usually marked improvement.

"9th. Usually, by the sixth day all desire for opium is gone. The patient then requires a change of air and surroundings, and tonics for a few weeks.

"The above is a rough outline of our treatment. Each case treated may require some change from the above.

"I believe that ninety-nine out of one hundred can be cured, if the patient has the requisite grace and grit. Out of eight hundred cases there has only been one death, and that was caused, I think, by apoplexy, and not by opium."

---

SUBERINE FOR CHAPPED NIPPLES.—(*L' Union Médicale du Canada*, January, 1879.)

The treatment recommended by M. Brochard for fissured nipples is so simple that it deserves to be popularized. When chaps exist on the nipples, whatever their extent, the nipple should be washed with pure water, and then dried and dusted with suberine, which, as is known, is impalpable cork powder. The author has used it for several years, and prefers it to lycopodium, for infants, because it contains tannin, and besides is much cheaper. Over the suberine is placed a piece of gold-beater's skin, cut star-shaped, in the center of which several punctures are made with a fine needle. Every time the child is suckled, the suberine is washed of with water, and the gold-beater's skin replaced, the child drawing the milk through it without giving pain. When the child is done, the suberine is again applied as before, and so on.

